# Freezing of gait in Parkinson's disease: disturbances in automaticity and control

**DOI:** 10.3389/fnhum.2012.00356

**Published:** 2013-01-10

**Authors:** Jochen Vandenbossche, N. Deroost, E. Soetens, D. Coomans, J. Spildooren, S. Vercruysse, A. Nieuwboer, E. Kerckhofs

**Affiliations:** ^1^Cognitive Psychology, Vrije Universiteit BrusselBrussels, Belgium; ^2^Neurological Rehabilitation, Vrije Universiteit BrusselBrussels, Belgium; ^3^Center for Neurosciences, Vrije Universiteit BrusselBrussels, Belgium; ^4^Department of Rehabilitation Sciences, Katholieke Universiteit LeuvenLeuven, Belgium

**Keywords:** Parkinson's disease, freezing of gait, cognitive control, automaticity, executive dysfunction

## Abstract

Recent studies emphasize a key role of controlled operations, such as set-shifting and inhibition, in the occurrence of freezing of gait (FOG) in Parkinson's disease (PD). However, FOG can also be characterized as a de-automatization disorder, showing impairments in both the execution and acquisition of automaticity. The observed deficits in automaticity and executive functioning indicate that both processes are malfunctioning in freezers. Therefore, to explain FOG from a cognitive-based perspective, we present a model describing the pathways involved in automatic and controlled processes prior to a FOG episode. Crucially, we focus on disturbances in automaticity and control, regulated by the frontostriatal circuitry. In complex situations, non-freezing PD patients may compensate for deficits in automaticity by switching to increased cognitive control. However, as both automatic and controlled processes are more severely impaired in freezers, this hampers cognitive compensation in FOG, resulting in a potential breakdown. Future directions for cognitive rehabilitation are proposed, based on the cognitive model we put forward.

Parkinson's disease (PD) is a progressive and neurodegenerative disorder of the nervous system. It is characterized by a substantial loss of dopaminergic cells in the substantia nigra pars compacta (Jankovic, 2008), although recent studies also point to an important contribution of non-dopaminergic degeneration (e.g., Rochester et al., 2012). PD can be associated with severe motor difficulties (tremor, rigidity, postural instability, and bradykinesia; Parkinson, 2002) and executive dysfunction (Lewis et al., 2003). Although not occurring in all patients, freezing of gait (FOG) can be seen as an independent feature of parkinsonism (Bartels et al., 2003). Nutt et al. (2011) defined FOG as “a brief, episodic absence or marked reduction of forward progression of the feet despite the intention to walk,” and is often described by patients as if their feet are glued to the floor for a short and transient period of time (Giladi et al., 1992). FOG episodes are not limited to gait alone, but can also occur in the upper limb (Nieuwboer et al., 2009; Vercruysse et al., 2012b). Knowledge about the processes causing this phenomenon is limited, and studies examining cognitive deficits related to FOG are scarce. Research investigating cognition in FOG by means of a dual task paradigm (e.g., walking while performing a secondary cognitive task), neuropsychological tests (e.g., SCOPA-COG), and cognitive experiments [e.g., Attention Network Task (ANT)], all point to a global cognitive decline and specific deficits (for a review, see Heremans et al., in press).

Giladi and Hausdorff (2006) described three categories of events that are prone to increase the occurrence of FOG episodes: (1) motor-based events (advanced PD motor symptoms like a disordered step control), (2) affective states (depression and/or anxiety), and (3) cognitive aspects (dual task). Vercruysse et al. (2012a) identified four independent risk factors contributing to the occurrence of a FOG episode: falls and balance problems, non-gait freezing, increased dopaminergic drug dose, and cognitive deficits. This emphasizes the wide range of triggers associated with FOG.

In an attempt to unravel the underlying mechanisms of FOG, Lewis and Barker (2009) proposed a pathophysiological model linking freezing episodes to motor, limbic, and cognitive brain loops. In this model, dopamine depletion results in an excessive synchronization of the output nuclei in the substantia nigra to the thalamus and pedunculopontine nucleus (PPN). The thalamus is involved in upstream pathways to cortical brain areas, important for regulation of behavior. The PPN is one of the major nuclei of the mesencephalic locomotor region, interconnected with basal ganglia and brainstem nuclei, vital for dynamic gait control including gait initiation, turning, stopping, avoiding obstacles, and adapting locomotion to the person's goals (Mena-Segovia et al., 2004). According to Lewis and Barker (2009), the inhibition on both thalamus and PPN has repercussions on motor, cognitive, and limbic circuits. Increased limbic (stress or anxiety) and sensory demands operate via a depleted dopaminergic system to the region of the caudate nucleus, resulting in sudden and intense episodes of excessive synchronization of the output nuclei on thalamus and PPN, hereby triggering FOG.

Investigating the cognitive abilities in PD and FOG is crucial for understanding basal ganglia functions and enhancing cognitive rehabilitation strategies. Nutt et al. (2011) postulated several hypotheses for the pathogenesis of FOG, two of which are particularly relevant for this viewpoint. First, an exaggerated loss of automaticity may explain why freezers have more difficulties with gait performance under dual task conditions (Spildooren et al., 2010) and why they benefit from external cues to drive their stepping pattern (Rahman et al., 2008). At the same time, it is possible that frontal executive dysfunction may evoke FOG episodes.

We conjecture that disturbances in controlled processes and automaticity are both important to understand the pathogenesis of FOG. Controlled processing (or executive control) entails the maintenance and stabilization of goal representations in working or prospective memory and the flexibility to update these goal representations when necessary (Cools, 2008). In contrast, the ability to perform a task without the need for executive control is referred to as “automaticity” (Posner and Snyder, 1975). It is important to note that from a pure process-based perspective, motor behavior always coincides with cognition (e.g., selecting, programming, and executing the proper motor response). Therefore, when we refer to cognitive dysfunction in freezers, we assume that this has an impact on both motor and non-motor aspects of their functioning. In the present review we depart from an in-depth analysis of controlled and automatic cognitive processes in the occurrence of freezing episodes. Subsequently, in order to explain FOG from a cognitive-based perspective, we will present a model mapping the disturbances in automaticity and executive control, and discuss the consequence for compensation strategies in freezers.

## Controlled processes and FOG

Cognitive deficits in PD predominantly reflect executive dysfunction, likely associated with the disruption of the frontostriatal circuitry (Lewis et al., 2003). Executive functioning refers to a collection of abilities located in the frontal lobe, sharing a common attribute (maintaining goal and context information in working memory), and encompasses three major functions. These functions are (1) shifting or switching between mental sets or tasks, (2) updating and monitoring of working memory contents, and (3) inhibition of prepotent responses (Miyake et al., 2000). Three neural structures are assumed to be important for executive functioning: the anterior cingulate gyrus, the dorsolateral prefrontal cortex, and the orbital frontal cortex (Chan et al., 2008). Disrupted projections between the frontal areas and the striatum are presumed to be associated with cognitive deficits in PD in general (Owen, 2004). For example, recent neuroimaging studies showed structural brain differences between freezers and non-freezers. Freezers exhibited (1) reduced functional brain connectivity within regions of the right fronto-parietal and the visual network (resting state fMRI; Tessitore et al., 2012b), (2) left parietal, occipital, and posterior cingulated cortex atrophy (Tessitore et al., 2012a), and (3) reduced activity in mesial frontal and posterior parietal regions (Snijders et al., 2011).

Neuropsychological assessment has been frequently used in order to pinpoint deficits in executive functioning in PD patients. However, for freezers this has been done less consistently. Several studies indicated that patients with FOG show dysfunctions in executive control compared to non-freezing PD patients. Amboni et al. (2008), for example, found that freezers exhibited a generalized executive dysfunction (Frontal Assessment Battery), cognitive inflexibility (Controlled Oral Word Association Test), and impaired inhibition (Stroop task). A follow up study, performed with the same group of subjects, showed that FOG was positively correlated with a deterioration of cognitive functions compared to non-freezers, whose cognitive status remained unchanged over time (Amboni et al., 2010). In another study, freezers also experienced set-shifting difficulties under temporal pressure, as measured by the Trail-Making-Test (Naismith et al., 2010). These differences also correlate with severity of FOG (Shine et al., 2012).

Compared to neuropsychological assessment, computerized cognitive tests have the advantage of being more sensitive to subtle differences in cognitive functions between patient groups. Executive control can be investigated by administering a well-validated experimental paradigm as the ANT (Fan et al., 2002). The ANT is a choice reaction time task developed to reliably dissociate three attention networks, namely alerting, orienting, and executive control on the basis of reaction time differences. Reaction time analysis allows determining impairment specific to the corresponding network. In our ANT study, we demonstrated that the ability to inhibit an unwanted response is impaired in both medicated and non-medicated freezers (Vandenbossche et al., 2011). Particularly, FOG patients seem to rely more on reflex-like behavior when they are confronted with conflicting stimuli compared to non-freezers and healthy controls. In complex situations this inevitably leads to more errors and slower responses (Vandenbossche et al., 2012). The knowledge that freezers revert to erroneous reflex-like behavior, instead of adjusted controlled actions in complex cognitive situations, denotes a specific deficit in inhibition. More particularly, freezers rely more on reflex-like responses as a result from their inability to inhibit irrelevant information. However, these reflex-like responses are also maladapted, leading to an error.

## Automaticity and FOG

Automaticity can be described as a process which occurs effortless, unconscious, and involuntarily (Posner and Snyder, 1975). Although automaticity deficits are assumed to play an important role in FOG (Wu and Hallett, 2005; Nutt et al., 2011), studies actually investigating this hypothesis are surprisingly scarce, and only focus on the *execution* of well-known automatic behavior, such as gait. The general assumption is that, if gait parameters are hampered under dual task conditions (a secondary cognitive task), *execution* of the behavior is no longer automatic (Hallett, 2008). Indeed, previous studies focusing on the *execution* of automaticity demonstrated that gait is affected under dual task conditions in PD (O'Shea et al., 2002; Hausdorff et al., 2003), and even more in FOG (Hackney and Earhart, 2010; Spildooren et al., 2010).

Studies investigating the *acquisition* rather than the *execution* of automaticity are even more scarce. The *acquisition* of automaticity can be examined in a controlled environment by use of a procedural learning task, like the serial reaction time task (SRT task; Nissen and Bullemer, 1987). The SRT task is a computerized reaction time experiment where subjects incidentally learn a repeating sequence of stimuli. Importantly, procedural learning as observed in the SRT task, is also achieved in healthy subjects when task performance takes place under high perceptual load (Deroost et al., 2009, 2012; Coomans et al., 2011), or attentional capacity (Jiménez and Vázquez, 2005). This supports the automatic nature of procedural learning. Interestingly, Poldrack et al. (2005) showed that for healthy young adults more emphasis on automaticity, and consequently less on cognitive control, was demonstrated by the end of the SRT task, as could be derived from reduced activity in frontostriatal circuits. Several studies show that sequence complexity, cognitive status, and stage of the disease are associated with procedural learning in PD patients in general (Deroost et al., 2006; Vandenbossche et al., 2009; Stephan et al., 2011). We recently found that PD patients suffering from FOG exhibit a specific impairment in procedural learning as measured by an SRT task (Vandenbossche et al., 2013). These results indicated that, under single task conditions, freezers demonstrated a reduced and weak learning effect compared to non-freezers and healthy controls. Although non-freezers were able to learn the sequence implicitly, a difference with healthy controls emerged. Importantly, this study also showed that under dual task conditions (by adding a secondary tone-counting task), non-freezers and healthy controls were still able to acquire procedural knowledge while freezers failed to do so.

## Interplay between automaticity and controlled cognitive processes

**Figure 1:**
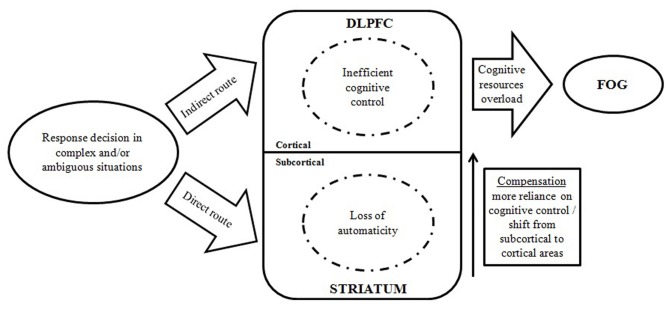
**Model demonstrating the interplay between automatic and controlled cognitive dysfunctions in the occurrence of FOG episodes.** (DLPFC, dorsolateral prefrontal cortex; FOG, freezing of gait).

In this part, we describe why the interplay between automaticity and cognitive control might be crucial in understanding FOG. Several studies (e.g., Spildooren et al., 2010) report that when task requirements are ambiguous or cognitively challenging, freezers seem to experience a complete breakdown in locomotor function, known as a FOG episode. These observations indicate that general cognitive resources are diminished in freezers compared to non-freezers. Lewis and Barker (2009) argued that an imbalance between motor, cognitive, and limbic activation is a key factor in explaining FOG episodes. Our aim is to analyze the role of specific processes in the occurrence of FOG. More specifically, we focus on the fragile balance between automaticity and control, regulated by the frontostriatal circuitry. To this extent, we constructed a model (see Figure 1) describing the disturbed cognitive pathway leading to a FOG episode, based on previous studies examining controlled operations and automaticity in freezers. Two tracks can be discerned: (1) a direct route requiring automatic responses regulated by the basal ganglia, and (2) an indirect route eliciting a controlled response regulated by frontal cortical areas. When automaticity and controlled processes are hampered, and cognitive resources are insufficient to handle a cognitively challenging situation, a FOG episode could possibly occur.

A key question is whether the loss of controlled cognitive processes (i.e., executive dysfunction) can be considered as an indirect effect of the loss of automaticity or alternatively whether both processes are simultaneously hampered in FOG. Although both automatic and controlled processing are presumed to be genuinely affected in PD in general (Koerts et al., 2009), and in FOG specifically (see “loss of automaticity” in the proposed model), interactions between both processes can still take place and can have a crucial influence on the occurrence of freezing episodes. For example, when confronted with a complex or ambiguous situation (e.g., dual tasking while turning), a sequence of both automatic and controlled cognitive action is needed to accomplish the task. In order to optimally coordinate these actions, efficient allocation of cognitive resources is needed. Since freezers, when compared to non-freezers, seem to be more impaired for both the *execution* (Hackney and Earhart, 2010; Spildooren et al., 2010) and the *acquisition* of automaticity (Vandenbossche et al., 2013), FOG can be described as a de-automatization disorder. Performing actions in an automatic manner spares cognitive resources for handling complex dual tasks. A loss of automaticity means that cognitive resources become increasingly pressured. As a consequence, a shift in neural activation from subcortical to more cortical areas (i.e., more reliance on controlled processes) can be expected as a compensation strategy (Redgrave et al., 2010). Yet, the increased load resulting from this compensation strategy, together with inefficient control processes in FOG (Vandenbossche et al., 2011, 2012), leads to an overload of cognitive resources, which in turn results in a breakdown, hence a FOG episode. Disturbances in automaticity and control are therefore crucial for understanding FOG and future research should focus on the specific interaction of these processes.

When unraveling underlying mechanisms of FOG, future studies also need to identify brain structures that have an impact on both motor events and cognitive aspects. A key structure in understanding FOG is possibly the PPN (Lewis and Barker, 2009). Although stimulation of the PPN showed variable results in the treatment of FOG (Thevathasan et al., 2011), the PPN is also part of the cholinergic pathway presuming to induce balance deficits and visuospatial and mnemonic deficits (Karachi et al., 2010; Kehagia et al., 2010). Imaging studies specifying the role of the PPN in the occurrence of FOG during execution of a sensitive computerized cognitive task are therefore indispensable.

## Future directions for cognitive rehabilitation

Given the specific impairments in the *acquisition* and *execution* of automaticity in freezers, and the interplay between automatic and controlled processes, cognitive therapies should tackle executive dysfunction, thereby increasing chances for successful compensation. Cognitive training, offered through interactive multimedia software and paper-and-pencil exercises stimulating both PD-specific (attention/working memory, memory, psychomotor speed, executive functions, and visuospatial abilities) and other cognitive domains, has already proven successful in PD patients (París et al., 2011). However, it remains crucial to determine whether freezers and non-freezers benefit from these therapies to the same extent. Rehabilitation therapy comprising both cognitive training and cueing techniques would greatly improve the quality of life of freezers by reducing the occurence of FOG episodes. Moreover, in analogy with motor rehabilitation, so-called *cognitive movement strategies* (Kamsma et al., 1995) can be very useful in alleviating cognitive deficits in PD and FOG. These strategies can ameliorate automaticity problems by focusing on explicit awareness of a complex action, and lower the relative pressure on controlled processes by defragmenting the action into smaller parts. Future studies should elucidate the underlying mechanisms of this effective movement therapy in an experimental setting, and consecutively investigate whether (1) it can be transferred to cognitive tasks, and (2) freezers show similar benefits compared to non-freezers.

## Conclusions

This viewpoint illustrates that the interplay between both automatic and controlled processes should be taken into account when investigating the underlying mechanisms of FOG. A cognitive model has been put forward to approach FOG from a cognitive-based perspective. We conclude that FOG can be seen as a multisystem disorder, in which episodes might evolve from disturbances in automaticity and controlled processing. Research investigating cognitive rehabilitation techniques strengthening cognitive compensation in freezers would strongly impact the quality of life of patients.

### Conflict of interest statement

The authors declare that the research was conducted in the absence of any commercial or financial relationships that could be construed as a potential conflict of interest.
